# Experimental Study on Drag Reduction Characteristics of Bionic Earthworm Self-Lubrication Surface

**DOI:** 10.1155/2019/4984756

**Published:** 2019-10-23

**Authors:** Guomin Liu, Xueqiao Wu, Meng Zou, Yuying Yan, Jianqiao Li

**Affiliations:** ^1^College of Civil Engineering, Jilin Jianzhu University, Changchun 130118, China; ^2^Faculty of Engineering, University of Nottingham, University Park, Nottingham NG7 2RD, UK; ^3^Key Laboratory of Bionic Engineering, Jilin University, Changchun 130022, China

## Abstract

In the present study, a coupling bionic method is used to study the drag reduction characteristics of corrugated surface with lubrication. In order to test the drag reduction features, bionic specimen was prepared inspired by earthworm surface and lubrication. Based on the reverse engineering method, nonsmooth curve of earthworm surface was extracted and the bionic corrugated sample was designed, and the position of lubrication hole was established by experimental testing. The lubricating drag reduction performance, the influence of normal pressure, the forward velocity, and the flow rate of lubricating fluid on the forward resistance of the bionic specimens were analyzed through a single factor test by using the self-developed test equipment. The model between the forward resistance and the three factors was established through the ternary quadratic regression test. The results show that the drag reduction effect is obvious, the drag reduction rate is 22.65% to 34.89%, and the forward resistance decreases with the increase of the forward velocity, increases with the increase of the normal pressure, and decreases first and then becomes stable with the increase of flow rate of lubricating fluid. There are secondary effects on forward resistance by the three factors, and the influencing order is as follows: normal pressure>flow rate of lubricating fluid>forward velocity.

## 1. Introduction

Soil adhesion is a major problem in the field of ground transportation, excavation, and farming machinery. The adhesion of soil to soil-engaging components will seriously affect the working efficiency and quality of ground machinery, thus increasing energy consumption [1–4]. With the aggravation of energy and environmental crises, it is imperative to design and produce soil-engaging components with high efficiency and low consumption. Earthworm is a typical soil animal, which has an excellent function of reducing drag and removing soil in long-term evolution. It can also move freely in clayey soil and does not have clay [5, 6]. Since early 1990s, scholars at home and abroad have gradually carried out bionic research on reducing soil adhesion and scouring soil of earthworm. Li et al. [7] carried out the extraction of earthworm surface fluid and studied the adhesion reducing the mechanism of earthworm surface self-lubrication. Sun et al. [8] measured the surface potential of earthworms; Ren et al. [9, 10] put forward the surface electroosmosis technology according to the phenomenon of earthworm surface electroosmosis and applied it to soil-engaging components. In the late 1990s, Ren et al. [11] studied the surface flexibility of earthworms and their antiviscosity characteristics. After 2004, Sun et al. [12, 13] and Li et al. [14–16] have successively carried out biomimetic research on the viscosity reduction and drag reduction of earthworm's nonsmooth surface. Shi et al. [17] carried out a biomimetic study on earthworm telescopic motion. Zu and Yan [18, 19] used the lattice Boltzmann method to simulate the surface electroosmosis of earthworms and further studied the adhesion reducing the mechanism of earthworm surface electroosmosis. These studies mainly focus on the unit biomimetic of a certain aspect or a certain factor.

With the deepening of bionic research, researchers have found that the functions, which biologically adapt to the external environment, are not only the role of a single factor or the simple addition of multiple factors but also the results of synergistic action that a variety of interdependent, mutually affecting factors are coupled through a certain mechanism [20, 21]. The research shows that earthworm has an excellent function of viscosity reduction and drag reduction, which is the result of multifactor coupling action such as body surface structure, body surface flexibility, body surface electroosmosis, body surface lubrication, and its unique movement mode [22–25]. Based on the nonsmooth structure and the self-lubricating characteristics of earthworm surface, this paper discusses the drag reduction characteristics of earthworm by a dual-coupling biomimetic method, in order to provide a new theory and method for the design of soil-engaging components with high efficiency and low consumption.

## 2. Materials and Methods

### 2.1. Coupling Bionic Sample Design

**Figure 1:**
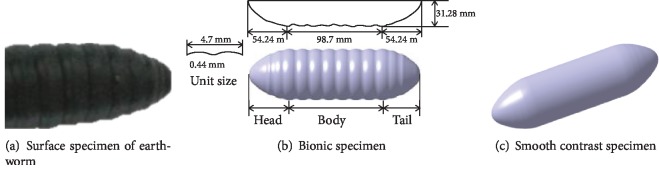
Specimen.

Earthworms are common animals that can move freely in the soil; it can adapt to different soil environments. The viscosity reduction ability of earthworms mainly depends on its flexible nonsmooth body surface, bioelectroosmotic system, surface lubrication, corrugated body surface, and other factors [12]. In this paper, *Eisenia foetida* was used as bionic prototype, and according to the previous studies, the corrugated body surface of earthworm has the characteristics of reducing viscosity and drag [21]. The effect of viscosity and drag reduction of the different states of the body from large to small is contraction state, motionlessness state, and stretched state, and the effect of viscosity and drag reduction of the head is more obvious than that of the body. Therefore, the head contraction state of earthworm has the best viscosity and drag reduction effect [14, 15], as shown in Figure 1(a).

Based on the reverse engineering method, the corrugated body surface of earthworm was scanned by a 3D laser scanner, and the data of point cloud was processed by Geomagic software. There are about ten knots involved in each extension-contraction movement of earthworms, the head and body are the main position of soil adhesion and force, and the tail are weak [14–16], so they are equivalent to a cylinder when designing biomimetic samples, composed of 10 body knots, and the tail curve is designed as the symmetrical curve of the head. Through the commands of three-point arc, tangent arc, and curve curvature extension in CAXA, the smooth curve which is close to the contour curve of the corrugated body surface of earthworm is drawn, and the coordinate point data of the smooth curve are extracted. Then, the spline curves of the head shape are drawn by using coordinate points. At last, amplifying the size by six times for engineering needs, the biomimetic sample was printed in 3D. As shown in Figure 1(b), the length is 207.18 mm, the width is 62.59 mm, the thickness is 31.28 mm, the sample material is photosensitive resin, and the printing precision is 0.1 mm. For comparative analysis, a smooth specimen of the same size was designed, as shown in Figure 1(c).

**Figure 2:**
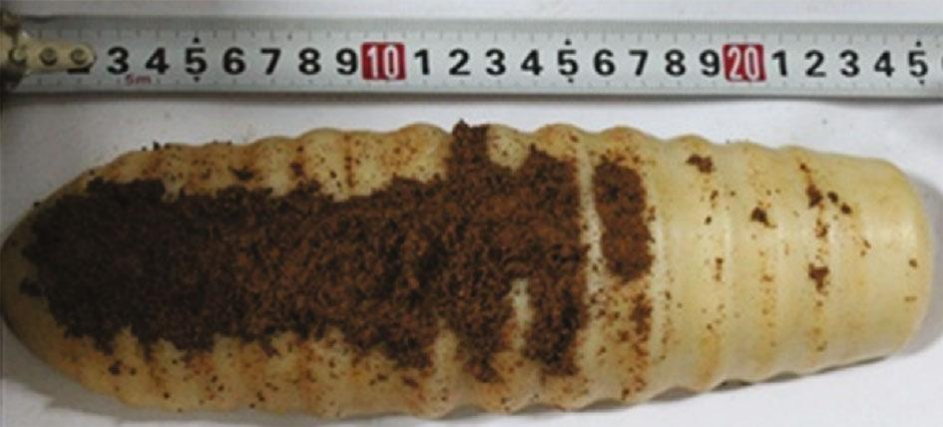
Adhesion when normal pressure was 34 N.

The lubrication hole's position of earthworm surface has direct influence on the lubricating effect [26], and the hole location of the corrugated surface sample is determined by the test method. According to previous studies, normal pressure is the most important factor affecting adhesion force. So, selecting the yellow clay of Changchun city, when the soil moisture content was 22.2% and the forward velocity was 200 cm/min, the experiment was carried out with five different normal pressure conditions, and each group was repeated three times. As shown in Figure 2, the adhesion was at a normal pressure of 34 N.

The adhesion data is shown in Table 1. According to Table 1, there is more adhesion to the head and body, but there is no adhesion in the tail.

**Figure 3:**
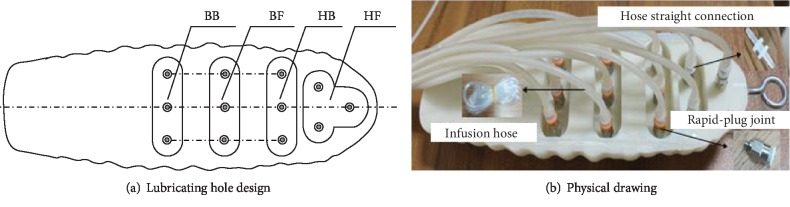
Coupling bionic specimen.

In order to prevent clogging, the bionic dorsal foramen was designed in the top of the sulcus of the head and body backward-forward direction according to the adhesion and model characteristics. They are including the anterior foramen of the head (HF), the posterior foramen of the head (HB), the anterior foramen of the body (BF), and the posterior foramen of the body (BB), as shown in Figure 3.

### 2.2. Test Rig and Methods

**Figure 4:**
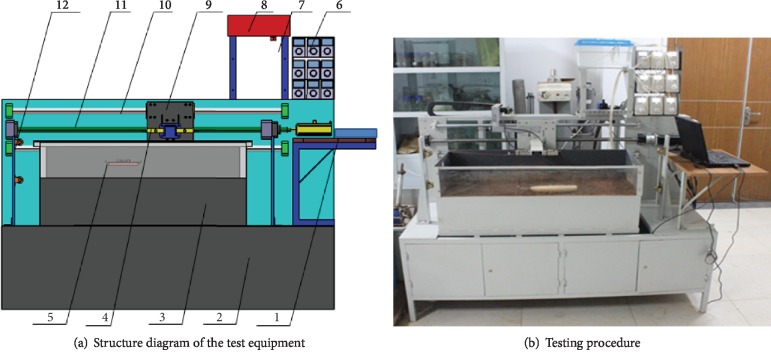
Test and equipment. 1: data acquisition device, 2: support frame, 3: soil bin, 4: force sensor, 5: bionic sample, 6: peristaltic pump, 7: lubricating adjustment device, 8: water tank, 9: moving plate, 10: motion control device, 11: screw drive set, 12: pulley block.

According to the test requirements, the test rig for lubricating and drag reduction performance of soil-engaging components was designed, as shown in Figure 4(a). The rig consists of support frame, soil bin, motion control device, data acquisition device, and lubrication adjustment device.

The soil is fixed on the support frame, and the motion control device is fixed directly above the soil bin, mainly including servomotor, pulley motion pair, and sliding plate, which is responsible for the bidirectional motion of the soil-engaging components in the soil bin. The lubrication adjustment device is fixed on the upper left side of the soil bin, mainly including peristaltic pump, peristaltic pump frame, and water tank, which can convey lubricating fluid during the movement of soil-engaging components. The pump is compiled by external software and connected to the computer. According to the conditions, the flow rate can be set and the number of the opening pump and the lubricating position can also be set, so that the flow rate can be accurately adjusted. The data acquisition device is fixed at the top of the soil bin, including computer, object board, and angle steel, which is responsible for collecting data and controlling each device compiled by software.

After the equipment connection is completed, the bionic sample is placed in the soil bin and the moving speed, the flow rate, and the number and position of the peristaltic pump are set up by the control software, and then the motor is started. The lead screw moves the bionic sample forward, and the data acquisition is transmitted to the computer by the data collector in actual time. The force sensor is Kistler Triaxial Force Sensor Type 9027C and assembly located and fixed between the traction rope and moving plate. The test image and the data can be obtained by software. For the testing procedure, see Figure 4(b).

## 3. Test and Result Analysis

### 3.1. Single Factor Test and Result Analysis

Forward velocity, flow rate of lubricating fluid, and normal pressure are important factors affecting forward resistance. In order to investigate their effects, a single factor test was carried out when soil moisture content was 22.2% with lubrication and nonlubrication, and three repeated tests were carried out to reduce the error. The test plan is shown in Table 2.

**Figure 5:**
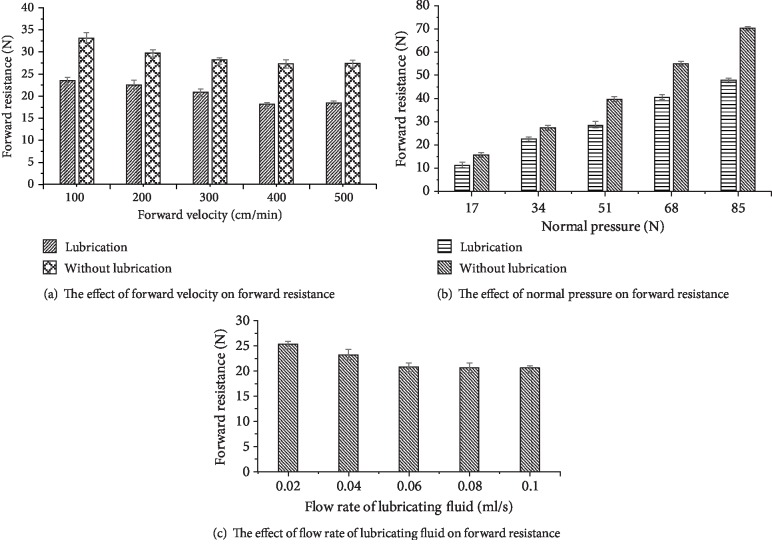
Results of a single factor test.

The effect of forward velocity on forward resistance is shown in Figure 5(a) when the normal pressure is 34 N and the flow rate of lubricating fluid is 0.06 ml/s. From Figure 5(a), it can be seen that the forward resistance is between 26.32 N~33.14 N without lubrication and 18.11 N~23.46 N with lubrication, and the drag reduction rate is 24.19% to 33.71%. In addition, the forward resistance decreases with the increase of the forward speed under both lubricated and nonlubricated conditions. Without lubrication, it decreases obviously with the increase of velocity, because when the velocity is small, the contact time between corrugated body surface and soil becomes longer and the disturbance to the soil increases in the course of movement. With lubrication, the forward resistance of 500 cm/min is greater than that of 400 cm/min, which is due to that the fluid cannot penetrate the interface between corrugated surface and soil sufficiently with the increase of velocity, resulting in a reduced lubrication effect.

When the forward velocity is 300 cm/min, and the flow rate of lubricating fluid is 0.06ml/s, the effect of normal pressure on the forward resistance is shown in Figure 5(b). From Figure 5(b), it can be seen that the forward resistance is 18.01 N~70.08 N without lubrication and 11.23 N~48.47 N with lubrication, and the drag reduction rate is 36.27%~58.46%. With the increase of normal pressure, the reducing effect is more and more obvious, and the forward resistance of unlubricated and lubricated conditions will increase continuously, because the contact area between corrugated surface and soil increases with the increase of normal pressure, and the gap between corrugated surface and soil decreases, which make soil compacted and the friction resistance increased.

When the normal pressure is 34 N and the forward velocity is 300 cm/min, the effect of the flow rate of lubricating fluid on the forward resistance is shown in Figure 5(c). From Figure 5(c), it can be seen that the forward resistance decreases first and then remains unchanged with the increase of the flow rate of the lubricating fluid. When the velocity rate is 0.08 ml/s, the forward resistance is 21.32 N that is close to 20.09 N when the rate is 0.1 ml/s. This is because with the increase of the flow rate of the lubricating fluid, the fluid fully permeates into the contact interface between corrugated surface and soil and then forms the interfacial lubrication, making the resistance decrease. When it increases to a certain condition, the interface water film is saturated, so the forward resistance is almost unchanged.

### 3.2. Ternary Quadratic Regression Combination Test and Result Analysis

In order to further explore the effect of these three factors and establish the equation between forward resistance and each factor, the three-element quadratic regression combination test [27] was carried out, and the repeated test with *m*_0_ = 3, *r*^2^ = 1.831 was selected. The factor level and coding are shown in Table 3.

According to the testing requirements and the designing principle of quadratic regression orthogonal, the experimental scheme is worked out and the regression coefficients and squares of each sequence are calculated as shown in Table 4.

By testing the regression coefficient, the equation is obtained as follows:

(1) $$\begin{array}{c} \hat{y}=16.10+5.68x_{1}-0.81x_{2}-1.29x_{3}+1.61{x_{1}}^{2}-0.96{x_{2}}^{2}-0.23{x_{3}}^{2}. \end{array}$$

Therefore, the sum of regressing squares is

(2) $$\begin{array}{c} S_{回}=S_{x_{1}}+S_{x_{2}}+S_{x_{3}}+S_{x_{1}^{\prime }}+S_{x_{2}^{\prime }}+S_{x_{3}^{\prime }}=287.69,\\ f_{回}=6. \end{array}$$

Because

(3) $$\begin{array}{c} S=\overset{13}{\underset{i=1}{\sum }}y_{i}^{2}-\frac{1}{13}{\left(\overset{17}{\underset{i=1}{\sum }}y_{i}\right)}^{2}=293.24, \end{array}$$

(4) $$\begin{array}{c} s_{R}=s-s_{回}=5.55, \end{array}$$

(5) $$\begin{array}{c} s_{e}=\overset{17}{\underset{i=1}{\sum }}\left(y_{i0}-\overset{¯}{y_{0}}\right)=0.93, \end{array}$$

(6) $$\begin{array}{c} s_{e}=\overset{17}{\underset{i=1}{\sum }}\left(y_{i0}-\overset{¯}{y_{0}}\right)=0.93, \end{array}$$

So

(7) $$\begin{array}{c} F_{回}=\frac{s_{回}/f_{回}}{s_{R}/f_{R}}=51.83>F\left(6,6\right)=8.47, \end{array}$$

(8) $$\begin{array}{c} F_{lf}=\frac{s_{lf}/f_{lf}}{s_{e}/f_{e}}=2.97<F_{0.25}\left(4,2\right)=3.23. \end{array}$$

The statistical test results show that the significant level of the equation is 0.01 and the fitting is very good, which can be considered as the best regression equation. The central processing and coding formula of the factors in the table are put into equation (1) and the regression equation is obtained as follows:

(9) $$\begin{array}{c} y=12.21+0.2z_{1}+100.98z_{2}-0.0028z_{3}+0.0093z_{1}^{2}-1066.45z_{2}^{2}+8.28\times 10^{-6}z_{3}^{2}. \end{array}$$

By testing the regression coefficient of formula (1), it is found that the order of the test factors affecting the forward resistance is as follows: normal pressure>flow rate of lubricating fluid>forward velocity, and the three factors have a significant effect on the forward resistance.

**Figure 6:**
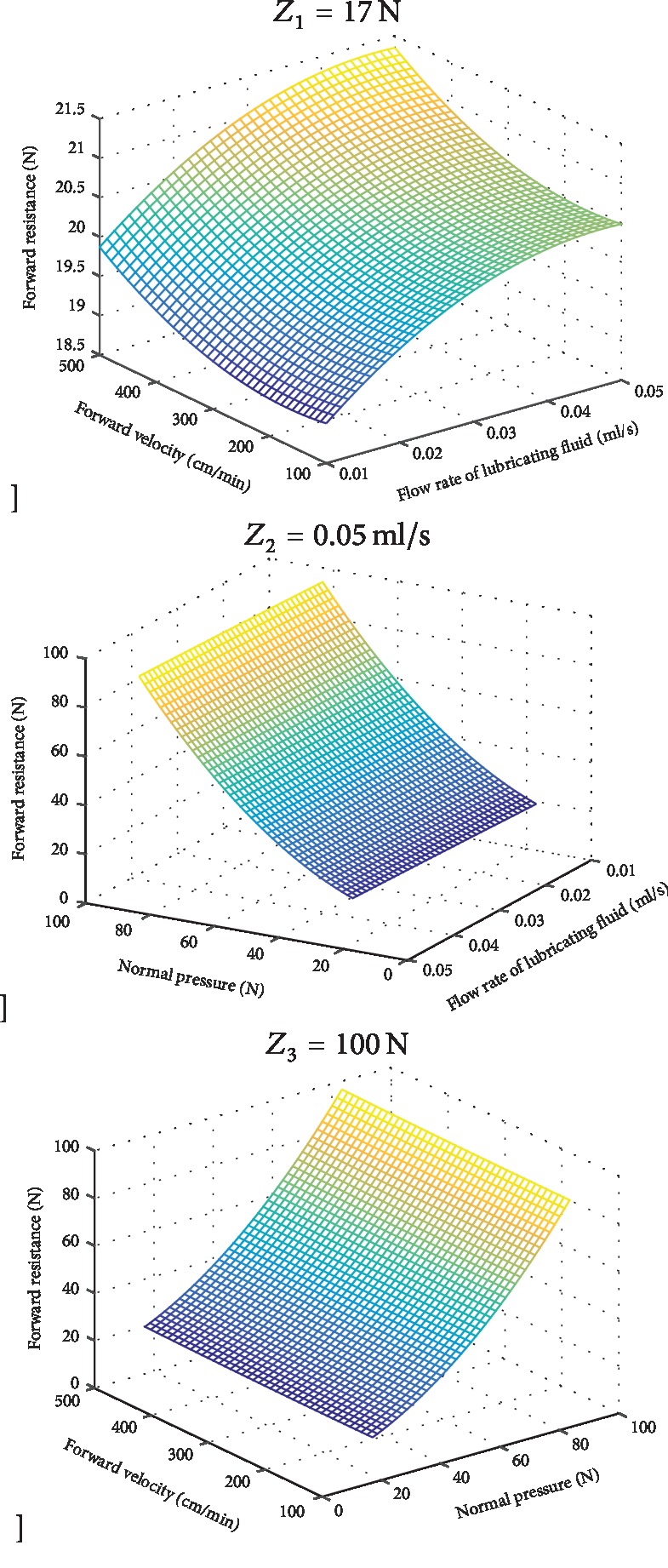
Results of orthogonal rotation combination test.

From formula (9), we can see that normal pressure *Z*_1_, flow rate of lubricating fluid *Z*_2_, and forward velocity *Z*_3_ have a secondary effect on the forward resistance, but there is no interaction between them, as shown in Figure 6.

## 4. Conclusion

We can conclude the following:

1. Based on the reverse engineering method, earthworm corrugated curves are extracted; bionic samples matched with real earthworm's body surface are designed. The bionic sample was printed by 3D printing, the location of lubricating hole was determined by the experiment method, and the bionic coupling of corrugated self-lubrication was realized
2. A test rig was designed and developed to test the lubricating drag reduction performance of soil-engaging components, which can accurately control the flow rate of lubricating fluid and measure the forward resistance of the sample quickly
3. The results of a single factor test show that the drag reduction of the bionic coupling sample is obvious, the drag reduction rate is 22.65%-34.89%, and the forward resistance decreases with the increase of forward velocity, which is proportional to the normal pressure, and at the same time, when the flow rate of lubricating fluid increases gradually, the forward resistance decreases first and then tends to stabilize
4. The regression equations between forward resistance and normal pressure, flow rate of lubricating fluid, and the forward velocity of the bionic coupling sample were obtained by using the ternary quadratic regression combination test. The regression analysis shows that the three factors have secondary effects on the forward resistance, and the order is as follows: normal pressure>flow rate of lubricating fluid>forward velocity, but there was no interaction between them

## Figures and Tables

**Table 1:** Adhesive quality of different parts.

Normal pressure (N)	Head adhesion (g)	Body adhesion (g)	Tail adhesion (g)	Total adhesion (g)
17	7.4	6.53	0.29	14.22
34	13.83	6.95	0.09	20.87
51	16.21	9.58	0.22	26.01
68	19.36	13.17	0.46	32.99
85	28.02	14.44	0.12	42.58

**Table 2:** Single factor test scheme.

Level	Factors		
	Forward velocity (cm/min)	Flow rate of lubricating fluid (ml/s)	Normal pressure (N)
1	100	0.01	17
2	200	0.02	34
3	300	0.03	51
4	400	0.04	68
5	500	0.05	85

**Table 3:** Natural factor level and its coding table.

*x*_*j*_(*z*_*j*_)	*Z*_1_ (N)	*Z*_2_ (ml/s)	*Z*_3_ (cm/min)
*r*	51	0.1	500
1	46.56	0.09	447.82
0	34	0.06	300
1	21.44	0.03	152.18
-*r*	17	0.02	100
$\Delta_{j}=\frac{z_{2j}-z_{1j}}{2r}$	12.56	0.03	147.82
$x_{j}=\frac{z_{j}-z_{0j}}{\Delta_{j}}$	*x*_1_ = 0.076 × (*z*_1_ − 34)	*x*_2_ = 33.33 × (*z*_1_ − 0.06)	*x*_3_ = 0.006 × (*z*_1_ − 300)

Note: *Z*_1_ means normal pressure; *Z*_2_ means flow rate of lubricating fluid; *Z*_3_ means forward velocity.

**Table 4:** Orthogonal rotation combination test plan and results.

	*X*_0_	*X*_1_(*z*_1_)	*X*_2_(*z*_2_)	*X*_3_(*z*_3_)	*X*_1_^2^(*X*_1_′)	*X*_2_^2^(*X*_2_′)	*X*_3_^2^(*X*_3_′)	*Y*_*i*_
1	1	1	1	1	1	1	1	20.09
2	1	1	-1	-1	1	1	1	22.64
3	1	-1	1	-1	1	1	1	9.84
4	1	-1	-1	1	1	1	1	10.14
5	1	*r*	0	1	*r* ^2^	0	0	26.96
6	1	-*r*	0	0	*r* ^2^	0	0	11.6
7	1	0	*r*	0	0	*r* ^2^	0	13.65
8	1	0	-*r*	0	0	*r* ^2^	0	16.18
9	1	0	0	*r*	0	0	*r* ^2^	13.53
10	1	0	0	-*r*	0	0	*r* ^2^	18.78
11	1	0	0	0	0	0	0	15.35
12	1	0	0	0	0	0	0	15.21
13	1	0	0	0	0	0	0	15.39
*D*_*j*_	13	7.66	7.66	7.66	6.19	6.19	6.19	
*B*_*j*_	209.36	43.53	-6.27	-9.95	10	-5.98	-1.44	
*b*_*j*_	16.10	5.68	-0.81	-1.29	1.61	-0.96	-0.23	
*S*_*j*_	3371.66	247.37	5.13	12.92	16.16	5.78	0.33	
*F*_*j*_		27701.11	574.72	1447.32	1809.08	646.93	37.51	
*α*_*j*_		0.01	0.01	0.01	0.01	0.01	0.05	

## Data Availability

We have submitted the raw data used in our manuscript and the other supplementary date are shown in the attachment. Other researchers can access the data supporting the conclusions of the study. (1) The nature of the data is the source data of the image in the paper; (2) the data can be accessed on the submitting system or through email to zoumeng@jlu.edu.cn; (3) there are no restrictions on the data access.
